# Evaluation and optimization of differentiation conditions for human primary brown adipocytes

**DOI:** 10.1038/s41598-018-23700-z

**Published:** 2018-03-28

**Authors:** XingYun Wang, LiangHui You, XianWei Cui, Yun Li, Xing Wang, PengFei Xu, LiJun Zhu, Juan Wen, LingXia Pang, XiRong Guo, ChenBo Ji

**Affiliations:** 1 Nanjing Maternity and Child Health Care Institute, The Affiliated Obstetrics and Gynecology Hospital of Nanjing Medical University, Nanjing, 210004 China; 2 Nanjing Maternity and Child Health Care Institute, Nanjing Maternity and Child Health Care Hospital, Nanjing, 210004 China

## Abstract

As an effective way to improve energy expenditure, increasing the mass and activity of brown adipose tissue (BAT) has become a promising treatment for obesity and its associated disorders. Many efforts have been made to promote brown adipogenesis and increase the thermogenic capacity of brown adipose cells (BACs). The present culture schemes for human BAC differentiation are mostly derived from white adipocyte differentiation schemes. To solve this issue, we compared the adipogenic and thermogenic effects of various components on human BAC differentiation and optimized their concentrations as well as the culture time for BAC differentiation. In this study, we found that the induction factors did not show a dose-dependent promotion of brown adipogenesis or thermogenic capacity. The higher differentiation levels did not inevitably result in higher BAT-specific gene expression levels or increased β_3_-receptor agonist sensitivity. As an important element of culture medium, triiodothyronine was found to be essential for differentiation and metabolic property maintenance. Furthermore, compared with other reported methods, this protocol induced a specific intrinsic differentiation program. Our study provides not only an optimized method for human BAC differentiation but also a cell model with good differentiation and thermogenic capacity for brown adipose research.

## Introduction

The continuously rising prevalence of obesity^1,2^ and the excess risk of death^3^ associated with obesity demand efficient treatment strategies. Obesity derives from excessive energy intake exceeding energy expenditure and results in excessive lipid accumulation in white adipose tissue (WAT)^4^, which can occur through an increase in adipocyte volume (hypertrophy) and number (hyperplasia). Conversely, the energy expenditure effect could be mediated by the expression of mitochondrial uncoupling protein 1 (UCP1) in brown adipose tissue (BAT)^5^, which had long been considered to only be present in rodents and human infants. With the development of ^18^F-fluorodeoxyglucose positron emission tomography and computed tomography (PET-CT)^6–8^, functionally active BAT depots have been identified in adult humans, and the increased BAT mass or thermogenic activity of existing BAT has been demonstrated to be inversely correlated with the body mass index (BMI), adiposity and fasting plasma glucose level in adult humans^7,9^. Therefore, there is renewed interest in BAT regarding the potential to combat the current epidemic spread of obesity and the related metabolic disorders, such as diabetes, cardiovascular diseases and nonalcoholic fatty liver disease^10^.

Although the use of rodent cell models has greatly informed our current understanding of BAT function^5,11,12^, investigations in human BAT are significantly less advanced. However, there are many differences in brown adipose cells (BACs) between rodents and humans in terms of the anatomical distribution^7,8^, mRNA expression signature^13^, cellular morphology, thermogenic capacity and response to acute glucocorticoid administration^12^. Previous studies of human brown fat cells have mainly been based on adult human BAT from the neck or supraclavicular region^5^, whose mRNA signature and function closely resembles mouse beige fat, instead of classical BAT^13^. Few efforts have been made to build an adipogenic method for classical brown fat cells^14–16^. Therefore, the establishment of an optimized differentiation method is helpful to fully elucidate the biological characteristics and therapeutic potential of classical human BACs^17^.

An ideal *in vitro* human BAC model contributes to a better understanding of adipogenesis, metabolic function, and underlying regulatory mechanisms. However, the lack of a widely accepted and well-characterized human cell model has limited the discovery of novel mechanisms for human brown adipocyte differentiation and activation. Commonly used cell models in human BAT studies mainly consist of immortal BAC lines^18^, primary cultures of human BACs^19^, human pluripotent stem cells^20^, and mesenchymal stem cells^21^. As concluded in several reviews^22,23^, the PAZ6 cell line, the first available immortalized human BAC line, could represent a mixed brown/brite (browning of white) phenotype, making it unsuitable for determining the molecular mechanisms characterizing these two cell types. Human multipotent adipose-derived stem cells could potentially be a suitable model for studying the conversion from white mature adipocyte to brown-like adipocyte^24^. Importantly, infant interscapular BAT (iBAT) has higher expression levels of the brown adipocyte marker ZIC1 and lower expression levels of the beige adipocyte marker TBX1 than adult human supraclavicular BAT, suggesting a truly brown identity^15,25^. The use of primary cells for *in vitro* experiments is a desirable strategy because it most closely resembles the physiological conditions *in vivo*^26^.

Therefore, based on reported adipogenic methods, we further evaluated and optimized differentiation conditions for human primary brown adipocytes. Furthermore, we compared the differentiation rate and thermogenic capacity of the reported methods and our proposed program for brown pre-adipocyte differentiation. This optimized differentiation method could provide a valuable tool for both the validation of existing targets and the identification of novel targets of human BAT activation.

## Materials and Methods

### Ethics statement

**Figure 1:**
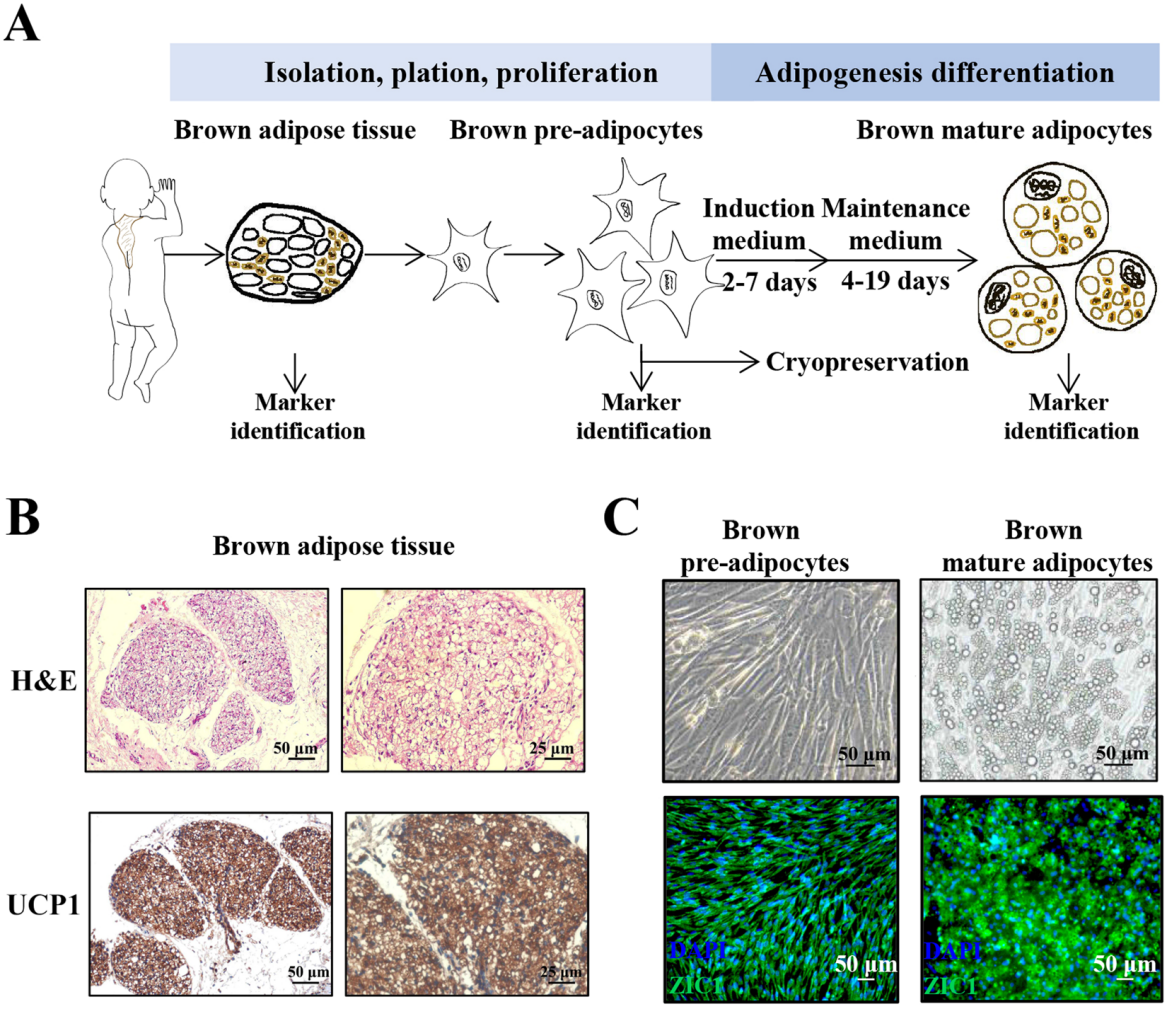
Isolation and identification of brown pre-adipocytes from interscapular brown adipose tissue (iBAT). (**A**) Schematic of procedure for the isolation, culture and differentiation of interscapular brown adipocytes (iBACs). The hallmarks (UCP1 and ZIC1) of iBACs were detected by immunohistochemistry (IHC) and immunofluorescence (IF) to detect the purity of the cells. Focusing on the induction period, we improved the components and the duration of induction time. (**B**) Hematoxylin and eosin staining of fetal interscapular adipose tissue (upper panel) revealed the presence of some dispersed cells with a multilocular aspect, characteristic of brown adipocytes. IHC staining of iBAT samples confirmed the positive result for the brown fat marker UCP1 (lower panel). Scale bar, 50 μm (left), 25 μm (right). (**C**) IF staining of ZIC1 on BACs before and after differentiation confirmed the purity of BACs. Scale bar, 50 μm.

All human fetal tissue was obtained from Nanjing Maternity and Child Health Care Hospital (Nanjing, China) from deceased donor and written informed consents were signed by parents. This study was approved by the medical ethics committee of Nanjing Maternity and Child Health Care Hospital (Permit number: [2015]110), and complies with The Population and Family Planning Law of the People’s Republic of China. In this study, a total of three samples were collected from the deep interscapular region of the deceased donors. All fetuses were spontaneously aborted (gestational age: 26^+5^, 26^+1^ and 26^+2^ weeks), and the sample collection process is depicted in Fig. 1A. In total, six tubes (approximately 6 × 10^6^ cells) were cryopreserved from each sample. The results of this experiment were verified using these three samples separately.

### Isolation, culture and differentiation of hBACs

As shown in Fig. 1A, BAT was minced and digested in DMEM/F12 (Life Technologies, CA, USA) containing collagenase II (1 mg/ml; Sigma-Aldrich, MO, USA) and fatty acid-free bovine serum albumin (BSA, 15 mg/ml; Sigma-Aldrich) for 20 min at 37 °C under gentle shaking. Following digestion, the suspension was filtered through a cell strainer (100 μm, Corning, NY, USA) and left to settle for 5 min. The layer below the floating mature adipocytes was aspirated using a syringe and passed through a thin filter (40 μm, Corning). The cell suspension was spun down and washed in DMEM/F12 by centrifugation for 10 min at 800 × g. The progenitors were resuspended in DMEM/F12 with 1% penicillin/streptomycin and 10% fetal bovine serum (FBS, Gibco, NJ, USA) and seeded in a 5-ml culture flask. The medium was changed the day after isolation and then every second day until the cells reached 80% confluence; then, the cells were split into 10-cm dishes and showed a well differentiation potential until six passages.

### RNA preparation and quantitative RT-PCR

Total RNA was isolated with TRIzol reagent (Invitrogen, CA, USA). To extract and purify total RNA, an RNeasy Mini Kit (Qiagen, Hilden, Germany) was applied in accordance with the manufacturer’s instructions. The concentration and purity of isolated RNA were evaluated using a NanoDrop 2000 system (Thermo Fisher Scientific, Waltham, MA, USA). A total of 1 µg of RNA from each sample was reverse transcribed into cDNA using a high-capacity cDNA reverse transcription kit (Applied Biosystems, Foster, CA, USA). Quantitative real-time PCR was carried out using a QuantStudio™ 7 Flex Real-Time PCR System (Applied Biosystems) according to the manufacturer’s instructions. The mRNA expression levels were determined using the 2^−△△Ct^ method. The results were normalized to peptidylprolyl isomerase A (PPIA) and are presented as the fold change of each gene. The primers used for RT-PCR were as follows: UCP1: sense sequence: 5′-AGGTCCAAGGTGAATGCCC-3′ and antisense sequence: 5′-TTACCACAGCGGTGATTGTTC-3′, peroxisome proliferator-activated receptor gamma coactivator 1 α (PGC1α): sense sequence: 5′-ACCTGACACAACACGGACAG-3′ and antisense sequence: 5′-GTCTCCATCATCCCGCAGAT-3′, fatty acid binding protein 4 (FABP4): sense sequence: 5′-GGCCAGGAATTTGACGAAGT-3′ and antisense sequence: 5′-ATCCCACAGAATGTTGTAGAGT-3′, cell death-inducing DFFA-like effector A (CIDEA): sense sequence: 5′-AGAGGTCGGGAATAGCGAGA-3′ and antisense sequence: 5′-GGATGTCGTAGGACACGGAG-3′, peroxisome proliferator-activated receptor γ (PPARγ): sense sequence: 5′-GCTGTGCAGGAGATCACAGA-3′ and antisense sequence: 5′-GGGCTCCATAAAGTCACCAA-3′, PPIA: sense sequence: 5′-TTCATCTGCACTGCCAAGAC-3′ and antisense sequence: 5′-TCGAGTTGTCCACAGTCAGC-3′.

### Protein extraction and western blot

Adipocyte cultures were rinsed once in ice-cold phosphate-buffered saline (PBS) and lysed in cell lysis buffer (20 mM Tris pH 7.5, 150 mM NaCl, 1 mM EGTA, 1 mM EDTA, 1 mM NaVO_4_, 1% Triton) with complete mini protease inhibitor cocktail (1 tablet/10 ml, Roche, CA, USA) and 1% phosphatase inhibitor cocktails 2 and 3 (Sigma-Aldrich). Protein concentrations were determined by the Bradford assay (Sigma-Aldrich) using BSA as a standard. Proteins were loaded on a 12% SDS-PAGE gel for electrophoresis, transferred to PVDF membranes, and immunoblotted with specific primary antibodies, as follows: rabbit polyclonal β-actin (Ab8227, Abcam, MO, USA; 1:5000 dilution), rabbit monoclonal UCP1 (Ab109483, Abcam, 1:1000 dilution), rabbit monoclonal FABP4 (Ab92501, Abcam, 1:1000 dilution) and rabbit monoclonal PPARγ (2435, Cell Signaling Technology, MA, USA; 1:1000 dilution). The secondary antibody was horseradish peroxidase-conjugated goat anti-rabbit IgG (1:5000 dilution) from Beijing Zhong Shan Biotechnology Co. (Beijing, China).

### Oil red O (ORO) staining

After the cells were fixed in 3.7% PBS-buffered formaldehyde for 1 h, they were stained with ORO solution (0.3% ORO in 60% isopropanol) for 2 h.

### Immunohistochemistry (IHC)

The BAT samples were placed in neutral-buffered formalin and processed routinely. After fixation for approximately 24 h, the tissue was then embedded in paraffin, sectioned manually and stained with hematoxylin and eosin (H&E). UCP1 was detected using rabbit polyclonal anti-UCP1 (Ab10983, Abcam, 1:500 dilution).

### Immunofluorescence

After washing with PBS twice, the adipocytes were fixed in 4% paraformaldehyde for 15 min and permeabilized with 0.25% Triton X-100 in PBS (PBST) for another 20 min. The fixed cells were maintained 2.5% BSA in PBST for 30 min at 37 °C and were then incubated with ZIC-1 antibody (Abcam, ab134951, 1:500 dilution) overnight at 4 °C. Then, the cells were incubated with the secondary antibodies conjugated with Alexa Fluor 488 (Invitrogen, 1:1000 dilution) for 1 hour at 37 °C. After the cells were washed with PBS three times, nuclei were stained with DAPI (Invitrogen, 1:10000 dilution) for 5 min. Finally, cells were visualized using a fluorescence microscope Imager A2 (Carl Zeiss, Werk Gottingen, Germany).

### Measurement of oxygen consumption

BACs were seeded in an X-24 cell culture plate (Seahorse Biosciences, MA, USA) coated with 0.1% galectin (Sigma-Aldrich). The cells were cultured and induced as described above. After the indicated days of differentiation, the medium was replaced with prewarmed, unbuffered measurement solution (DMEM basal medium (Sigma D5030) supplemented with 25 mM glucose, 2 mM sodium pyruvate, 31 mM NaCl, 2 mM GlutaMax and 15 mg/l phenol red, pH 7.4) with 2% fatty acid-free BSA and incubated at 37 °C in a room air incubator for 1 h, as described by Li Y^27^. Oxygen consumption rates (OCRs) were measured at basal levels followed by loading with drugs destroying the respiratory chain, including oligomycin (ATP synthase inhibitor, 1 μM), forskolin (1 μM), FCCP (uncoupling agent, 0.5 μM) and a rotenone/antimycin mixture (complex I and complex III inhibitor, 0.5 μM) using an XF Extracellular Flux analyzer (Seahorse Bioscience) according to the manufacturer’s instructions.

### Statistical analysis

All experiments were repeated at least three times and performed in triplicate. Statistical analysis was performed using Student’s t-test for comparisons between two groups, and one-way ANOVA was used for multiple comparisons. *P < 0.05, **P < 0.01 and ***P < 0.001 were considered significant. Data are shown as the mean, with error bars representing the standard deviation (S.D.) or standard error of the mean (S.E.M.).

## Results

### A comparison of differentiation cocktails for human BACs

As early as the 1980s, Cigolini *et al*.^28^ had succeeded in separating human primary adipose precursors from BAT depots, but it took decades to improve their differentiation conditions. Great advances in the modification of the differentiation of pre-adipocytes to brown or brown-like adipocytes have been achieved in the past several decades. Here, we summarize different brown adipogenic induction cocktails for brown adipocyte differentiation (Table 1). As shown in Table 1, various induction media and concentrations of critical induction factors, including insulin, 3-isobutyl-1-methylxanthine (IBMX), and dexamethasone (DEX), have been used. Some agents, including indomethacin (INDO)^29^ and triiodothyronine (T3)^30^, have been reported to exert a browning effect on white and beige adipocytes and were also selectively added to the induction formulation for hBACs. Moreover, in some studies^31^, apo-transferrin, pantothenate acid and d-biotin were also added to the differentiation formula to improve the differentiation of BACs. However, their roles in improving the adipogenic differentiation and thermogenic capacity of BACs remain undetermined.

**Table 1:** Differentiation cocktails for human brown fat cell models.

Group		Seiler SE *et al*.^16^		Shinoda K *et al*.^32^		Lee P *et al*.^19^		Jespersen NZ *et al*.^13^		Present study	
		a		b		c		d		e	
Sample source		Human fetal tissue		30–43 years adults		25–41 years adults		23–57 years adults		Human fetal tissue	
Differentiation cocktail		Induction cocktail	Maintenance cocktail	Induction cocktail	Maintenance cocktail	Induction cocktail	Maintenance cocktail	Induction cocktail	Maintenance cocktail	Induction cocktail	Maintenance cocktail
**Basic conditions**	**Medium**	DMEM (HG)	DMEM (HG)	DMEM/F12	DMEM/F12	DMEM/F12	DMEM/F12	DMEM/F12	DMEM/F12	DMEM/F12	DMEM/F12
	**FBS** ***(v/v)***	10%	10%	10%	10%	—	—	—	—	—	—
	**FCS** ***(v/v)***	—	—	—	—	10%	10%	—	—	—	—
**Basic components**	**Insulin**	850 nM	160 nM	860 nM	860 nM	850 nM	850 nM	100 nM	100 nM	430 nM	430 nM
	**IBMX**	0.25 mM	—	0.2 mM		0.5 mM	0.5 mM	0.54 mM	—	0.5 mM	—
	**DEX**	5 μM	—	5 μM	5 μM	1 μM	1 μM	0.1 μM	0.1 μM	1 μM	—
	**ROG**	1 μM	—	1 μM	—	1 μM	1 μM	0.2 μM	0.2 μM	1 μM	—
	**INDO**	100 μM	—	125 μM	—	—	—	—	—	—	—
	**T3**	1 nM	—	1 nM	1 nM	1 nM	1 nM	2 nM	2 nM	1 nM	1 nM
**Additional components**	**Apo-transferrin**	10 μg/ml	—	—	—	10 μg/ml	10 μg/ml	10 μg/ml	10 μg/ml	10 μg/ml	—
	**d-Biotin**	—	—	—	—	33 μM	33 μM	—	—	33 μM	—
	**Pantothenate**	—	—	—	—	17 μM	17 μM	—	—	17 μM	—
	**GH**	—	—	—	—	1 nM	1 nM	—	—	—	—
	**IGF-I**	—	—	—	—	1 nM	1 nM	—	—	—	—
**Time length**	**Differentiation days**	21 (7 for induction) days		21 (2 for induction) days		7 days		12 (3 for induction) days		8~10 (4 for induction) days	

**Figure 2:**
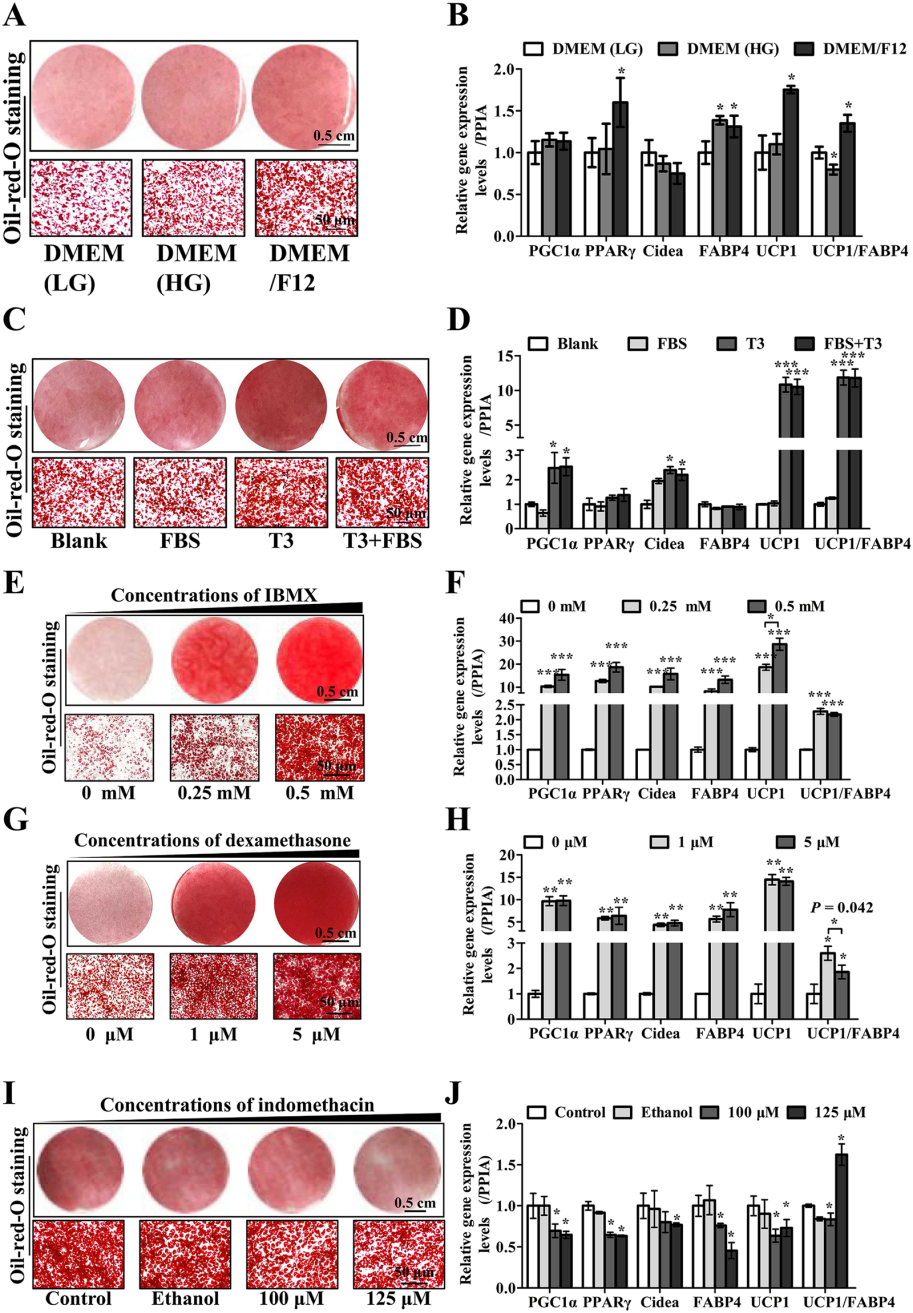
The effect of different basic components on adipogenic and thermogenic capacity during primary brown adipocyte induction. Representative images of Oil red O (ORO) staining and RT-PCR were carried out to assess the adipogenic levels of BACs by (**A**,**B**) differentiated media, (**C**,**D**) triiodothyronine (T3) and fetal bovine serum (FBS) in the culture media, (**E**,**F**) different concentrations of 3-isobutyl-1-methylxanthine (IBMX), (**G**,**H)** dexamethasone (DEX), (**G**,**H**) and indomethacin (INDO) (**I**,**J**) on adipogenic and thermogenic capacity, respectively. “Blank” group in Fig. 2A and “Control” group in 2I were consist of DMEM/F12, 860 nM insulin, 0.5 mM IBMX, 5 μM DEX, 1 μM ROG and 1 nM T3 (4 days for induction). Scale bar, 50 μm. PPIA was used as the internal control for RT-PCR. The ratio of UCP1-to-FABP4 expression was used to represent the browning level, and FABP4 served as an internal control to eliminate differences caused by the cell differentiation level. These experiments were repeated at least three times and performed in triplicate. Quantitative data are presented as the mean ± S.D. (n = 3). *P < 0.05; **P < 0.01; ***P < 0.001 compared with the control.

**Figure 3:**
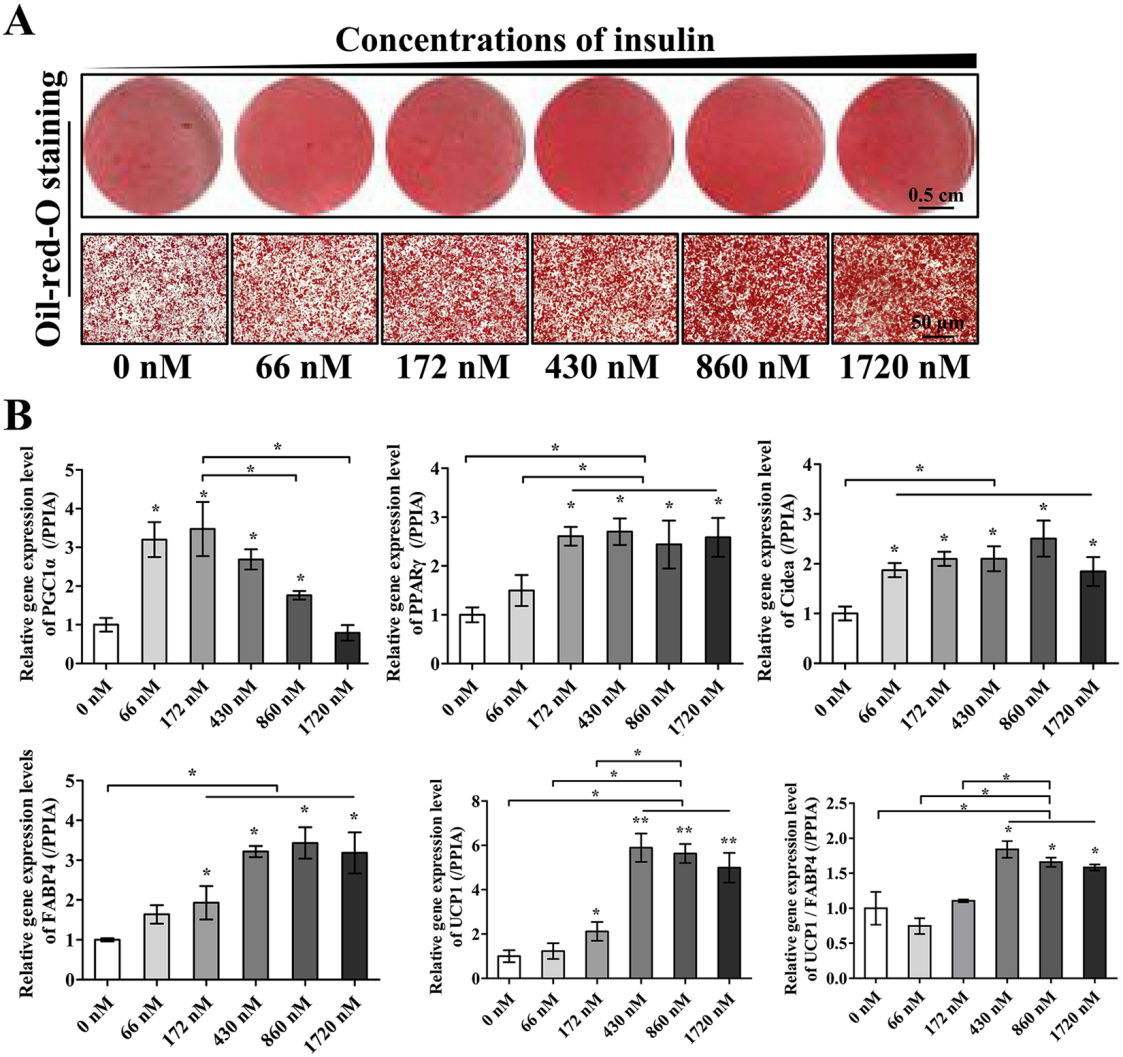
The effect of different insulin concentrations on adipogenesis and thermogenesis during BAC differentiation. (**A**) Representative images of ORO staining to indicate the level of adipogenesis under increasing insulin concentrations. Scale bar, 50 μm. (**B**) Adipogenic and thermogenic gene expression levels were measured by RT-PCR. PPIA was used as the internal control. All the experiments were repeated at least three times with similar results. *P < 0.05; **P < 0.01; multiple comparisons were analyzed by one-way ANOVA.

**Figure 4:**
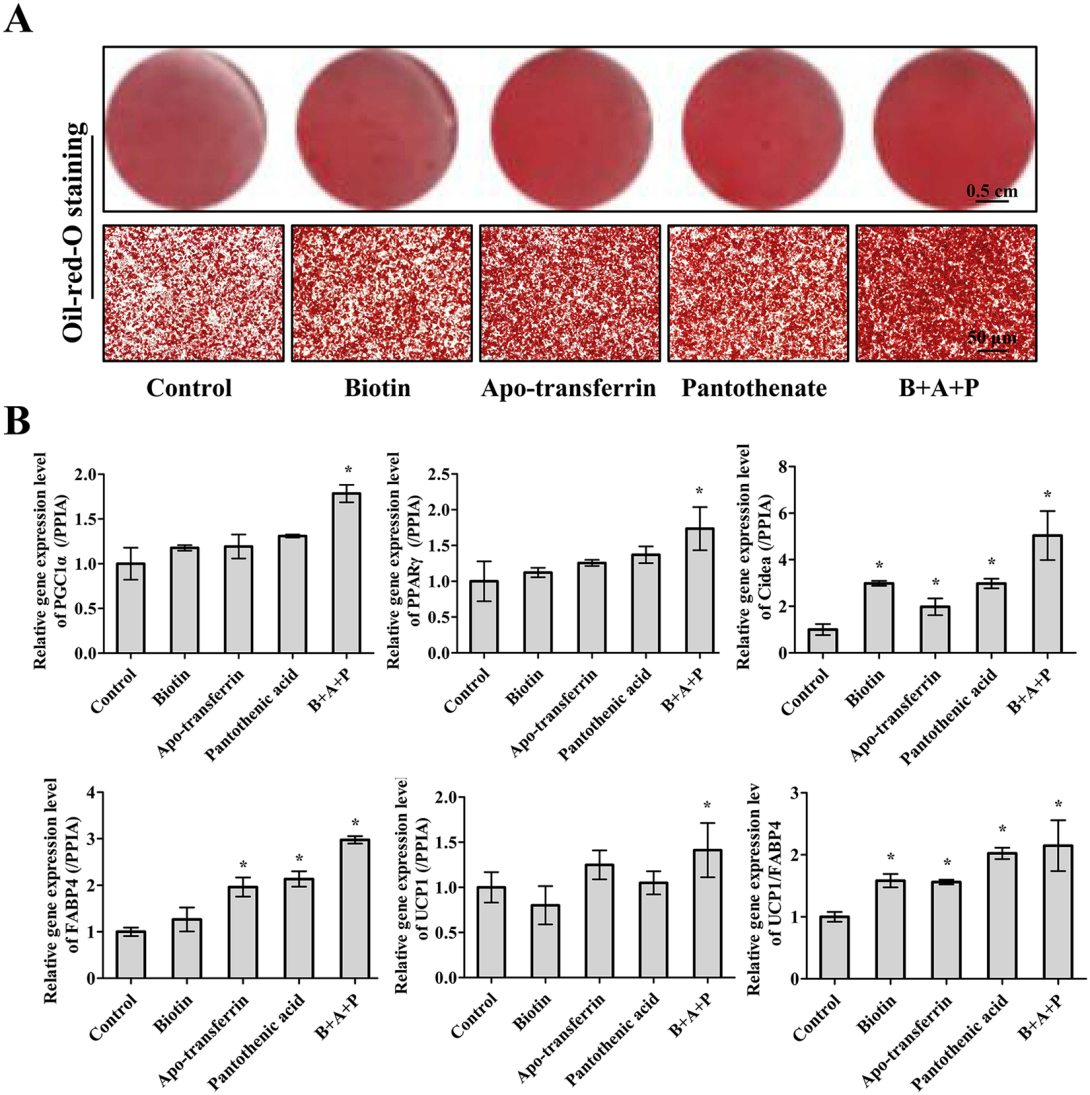
The promoted effect of additional supplements on brown adipocyte differentiation. The effects of pantothenate, d-biotin and apo-transferrin addition were assessed by (**A**) ORO staining on lipid droplet accumulation and (**B**) RT-PCR detection of browning and differentiation gene expression. PPIA was used as the internal control. Scale bar, 50 μm. Quantitative data are presented as the mean ± S.D. (n = 3). All the experiments were repeated at least three times with similar results. *P < 0.05 compared with the control.

**Figure 5:**
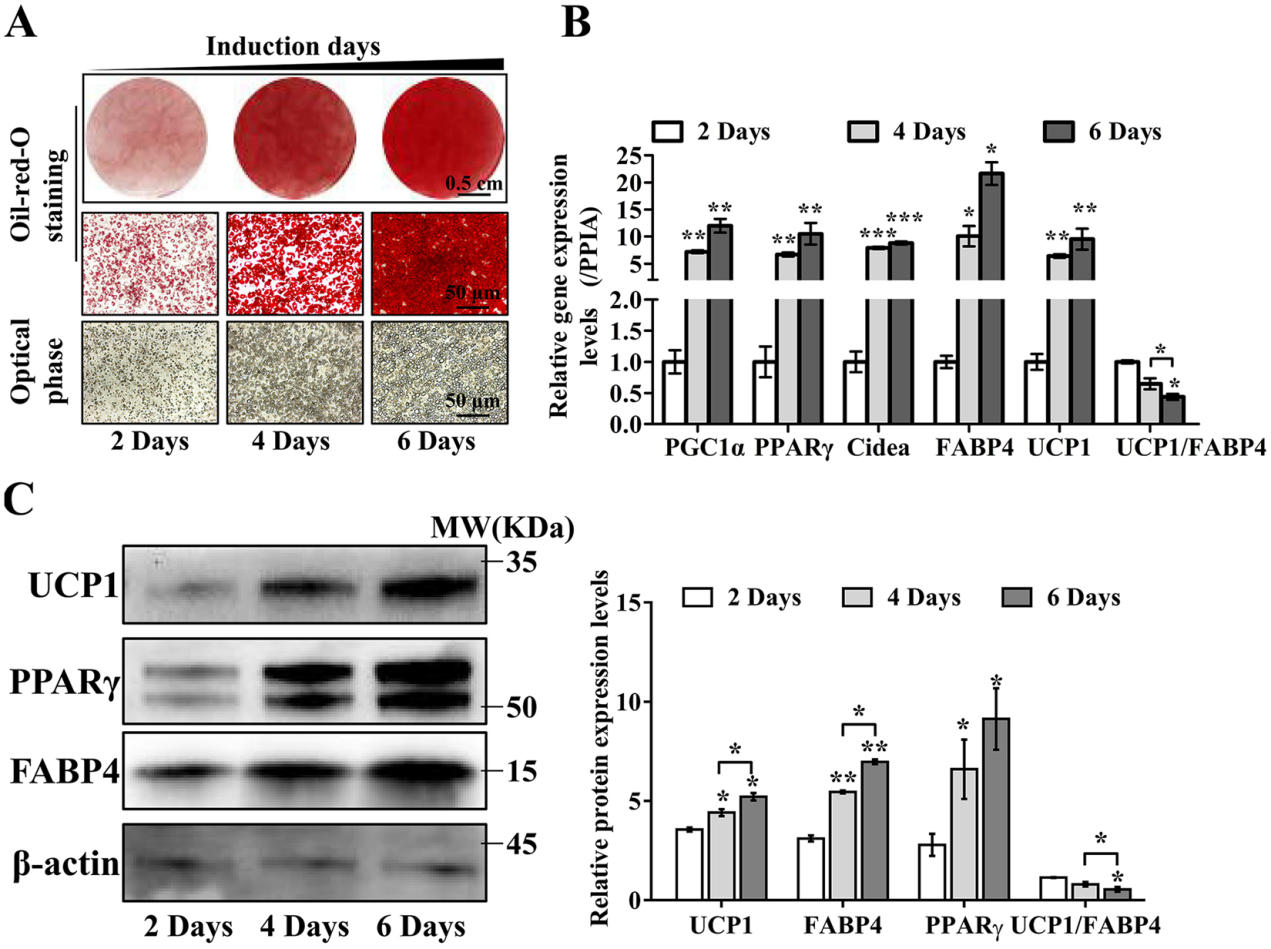
Comparison of the duration of induction on BAC differentiation. (**A**) ORO staining (upper panel) and phase-contrast images (lower panel) were used to evaluate differentiation after different induction durations (2, 4, and 6 days). Scale bar, 0.5 cm (upper panel) and 50 μm (middle and lower panels). (**B**) RT-PCR was used to detect adipogenic and thermogenic gene expression levels to assess the differentiation of BACs after different induction durations. PPIA was used as the internal control. Quantitative data are presented as the mean ± S.D. (n = 3). The experiments were repeated at least three times with similar results. (**C**) Representative western blot analysis of UCP1, PPARγ and FABP4 expression. The data were normalized to β-actin antibody and analyzed using ImageJ software in three repeated experiments. The protein expression levels of UCP1, PPARγ and FABP4 in the 4-day and 6-day groups are expressed as relative increases compared with the 2-day group. There was a decrease in the UCP1/FABP4 ratio in the 6-day group compared with the 4-day and 2-day groups. Data are presented as the mean ± S.D. *P < 0.05; **P < 0.01; ***P < 0.001 compared with the 2-day group.

**Figure 6:**
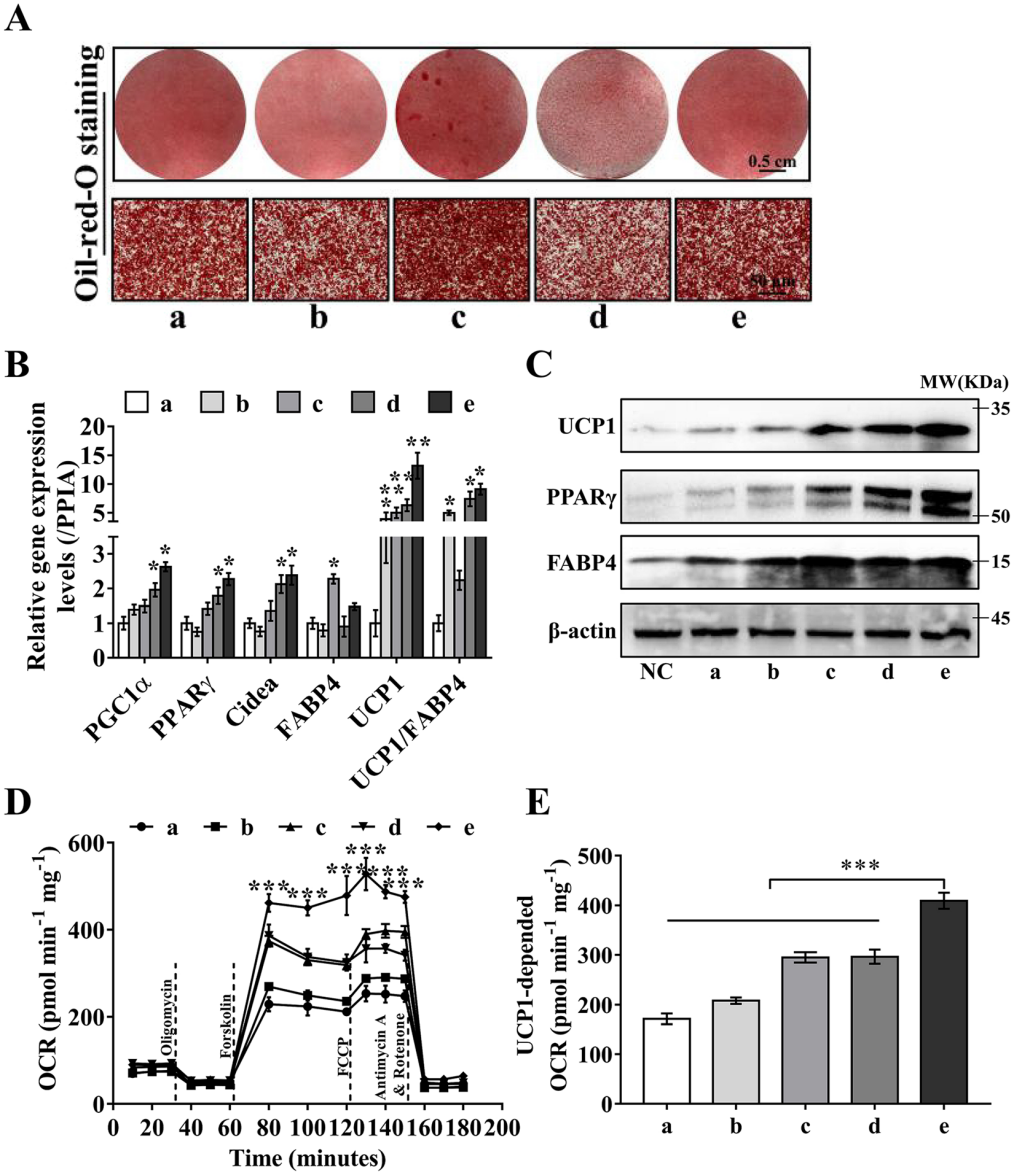
Comparison of the differentiation, browning and functional properties of BACs induced by different protocols. (**A**) ORO staining was performed to assess lipid accumulation in the different programs. Scale bar, 0.5 cm (upper panel) and 50 μm (lower panel). (**B**) RT-PCR was used to evaluate the expression levels of the adipogenic and thermogenic genes PGC1α, PPARγ, CIDEA, FABP4 and UCP1. PPIA was used as the internal control. (**C**) Protein expression levels of brown fat and adipogenic markers, including UCP1, PPARγ and FABP4, relative to β-actin expression in differentiated adipocytes. (**D**) Mitochondrial respiratory function or oxygen consumption rate (OCR) measured by a Seahorse XF24 Extracellular Flux Analyzer and normalized to the total protein concentration. Representative time course of OCRs of primary brown adipocytes inducted by different protocols measured in the presence of 2% BSA. BACs induced by protocol e presented the highest maximal OCR and UCP1-dependent OCR. Arrows indicate the administration of respiratory inhibitors, including oligomycin, forskolin, FCCP, and antimycin A plus rotenone. (**E**) Quantitation of the UCP1-dependent OCRs from part D. The experiments were performed in duplicate using cells derived from 3 different donors. Data are the mean ± S.E.M., *P < 0.05, **P < 0.01, ***P < 0.001. Statistical analysis was performed by Student’s t-test between two groups, and one-way ANOVA was used for multiple comparisons.

To establish a proper program based on the method by Shinoda K *et al*.^32^, we verified the basic induction cocktail consisting of basic media and the involved factors (T3, FBS, IBMX, DEX and INDO) of human BAT precursor differentiation (Figs 1 and 2). Then, based on the basic cocktail, we detected the appropriate concentration of insulin (Fig. 3). Furthermore, the need for additional ingredients (d-biotin, apo-transferrin and pantothenate acid) was estimated (Fig. 4). The length of induction (in days) also served as an important factor for BAC differentiation (Fig. 5). Compared with the reported induction programs for BACs, our program showed increased adipogenic and thermogenic capacity (Fig. 6). The evaluation indexes included thermoregulatory gene expression levels, adipogenic gene expression levels, lipid droplet accumulation, mitochondrial copies, OCRs and lipolysis viability.

### Isolation and identification of human brown pre-adipocytes

To isolate human brown adipose progenitor cells, BAT was collected from the interscapular region. At the same time, H&E staining and IHC for UCP1 were performed to validate the accuracy of BAT sample separation (Fig. 1B). The samples showed dispersed multilocular cells and strong UCP1 expression without clear evidence of unilocular white adipocytes. The immunophenotypical characterization of such cultures revealed strongly positive results for the brown adipocyte marker ZIC1 before (Fig. 1C, left) and after differentiation (Fig. 1C, right), suggesting that we obtained a final enrichment of a pure population of classic brown adipocytes^25^.

### Comparison of basic induction cocktail components

The adipogenic cocktail method has been commonly used to study various aspects of adipocyte biology and adipogenesis. We first detected the effects of different media and basic agents on hBAC adipogenic and thermogenic levels. The ratio of UCP1-to-FABP4 expression^33,34^ was used to represent the browning level, and FABP4 served as an internal control to eliminate differences caused by the cell differentiation level. The BACs induced in DMEM/F12 showed increased lipid droplet accumulation (Fig. 2A) and a significantly (P < 0.05) higher expression level of PPARγ, FABP4 and UCP1 compared with those induced in DMEM (low glucose, LG) (Fig. 2B). While the DMEM (high glucose, HG) group showed significantly increased FABP4 expression, the UCP1/FABP4 ratio dropped obviously, indicating that DMEM/F12 was a more appropriate induction medium for BACs than DMEM (LG) or DMEM (HG). Therefore, we determined the basic culture conditions and defined them as the “Blank” group (DMEM/F12, 860 nM insulin, 0.5 mM IBMX, 5 μM DEX, 1 μM ROG, 1 nM T3 and 4 days for induction) and continued to study other effects of induction factors.

Next, we examined the effects of FBS (10%, *v/v*, Gibco) and T3 on adipogenic and thermogenic differentiation in the same medium (DMEM/F12). Lipid droplet volume was robustly elevated by T3 stimulation (Fig. 2C). Moreover, T3 treatment significantly increased the mRNA expression levels of PGC1α (2.5-fold, P < 0.05), UCP1 (10-fold, P < 0.001) and UCP1/FABP4 (10-fold, P < 0.001) (Fig. 2D). A similar expression pattern was observed for other brown fat differentiation markers, such as PPARγ and CIDEA (Fig. 2D). However, not significant differences were found for FABP4. These results indicated that T3 could promote BAC thermogenic differentiation more than lipid droplet accumulation. Moreover, we found that while FBS could slightly promote differentiation, there were no significant differences compared with the control group.

Furthermore, we examined the effects of different concentrations of IBMX, DEX and INDO on adipogenic and thermogenic differentiation. Both 0.25 mM and 0.5 mM IBMX significantly increased the expression of thermogenic and adipogenic genes (8-, 28-fold), indicating the critical role of IBMX in promoting adipogenic differentiation in adipocytes. The 0.5 mM BAC group showed more effective differentiation than the 0.25 mM group (Fig. 2E,F), revealing a dose-dependent effect of IBMX.

Traditionally, the effects of DEX on adipocyte differentiation have been explained as glucocorticoids permitting the effects; however, some researchers have found that DEX could directly increase the levels of intracellular cyclic adenosine monophosphate (cAMP) and lipolysis in adipocytes^35^. In our study, DEX upregulated the expression of PGC1α (9.6-, 9.7-fold), PPARγ (5.8-, 6.3-fold), CIDEA (4.3-, 4.7-fold), FABP4 (5.6-, 7.7-fold), and UCP1 (14.47-, 14-fold) at concentrations of 1 μM and 5 μM (P < 0.01), respectively. Increasing the concentration of DEX to 5 μM did not significantly increase the expression of these genes. However, a decrease in the UCP1/FABP4 ratio was observed at a concentration of 5 μM compared with 1 μM, indicating that 1 μM DEX was sufficient for BAC induction (Fig. 2G,H).

INDO, a cyclooxygenase-2 inhibitor, is a typical culture addition for mouse pre-adipocyte differentiation to brown/beige adipocytes^36,37^ and sometimes for human brown adipocytes^12^. In this study, INDO significantly suppressed the expression of both FABP4 (0.76-, 0.45-fold, P < 0.05) and UCP1 (0.63-, 0.73-fold, P < 0.05) in the adipogenic cocktails (Fig. 2I,J). Thus, INDO might not be a suitable induction cocktail ingredient for hBACs.

Thus far, we determined that the basic cocktail consisted of DMEM/F12, 1 nM T3, 0.5 mM IBMX, 1 μM DEX and 1 μM ROG. Because they were clearly beneficial for BAC differentiation in our study.

### Effects of insulin concentration on human brown pre-adipocyte differentiation

Insulin is a potent adipogenic hormone that triggers a series of transcription factors governing differentiation from pre-adipocytes into mature adipocytes. However, the insulin concentration varies across a large number of adipogenic induction programs^38^. To identify the proper concentration for BAC induction, we tested concentrations from 0 nM to 1720 nM (0, 66, 172, 430, 860, 1720 nM). As the insulin concentration increased, lipid droplet accumulation increased steadily, as measured by ORO staining (Fig. 3A). Correspondingly, the qPCR results showed that the expression levels of common adipogenic genes (PPARγ and FABP4) were elevated significantly (Fig. 3B). In addition, the brown fat cell marker UCP1 increased significantly at high concentrations (66 nM, 172 nM, 430 nM, 860 nM and 1720 nM). Among these groups, the 430 nM group showed the highest UCP1 expression level. Accordingly, high insulin concentration groups (430 nM, 860 nM, 1720 nM) showed high levels of UCP1 and FABP4 expression, and the 430 nM group showed the highest UCP1/FABP4 (1.8-fold) ratio. Similarly, PGC1α expression was increased in the 66, 172, 430, and 860 nM groups, while the 860 nM group showed the highest CIDEA expression level.

Thus, we demonstrated that at concentrations ranging from 172–860 nM, insulin could promote not only adipogenesis but also brown fat gene expression appreciably. Furthermore, insulin concentrations higher than 430 nM could inhibit the expression of certain brown fat-specific genes. Insulin concentrations ranging from 430 nM to 860 were suitable for BAC induction, but the highest expression level of UCP1 was observed at a concentration of 430 nM.

### Effects of additional supplements on brown pre-adipocyte differentiation

Types of B vitamins, such as pantothenate acid and d-biotin, and apo-transferrin are common factors added to serum-free media for cell culture and differentiation, especially for adipocytes^39,40^. Moreover, d-biotin acts as a coenzyme for carboxylases regulating lipid and amino acid metabolism in adipose tissue^41^. The addition of pantothenate acid, d-biotin, and apo-transferrin increased lipid accumulation (Fig. 4A). Remarkably, the expression levels of both adipogenic and thermogenic genes were significantly improved (Fig. 4B).

### Effects of induction period length on hBAC differentiation

For adipose stromal cells, investigators have declared that extending the length of the induction period with an adipogenic cocktail improves the degree of differentiation and the metabolic phenotype^42^. In our study, we detected that a prolonged induction time could significantly increase lipid droplet accumulation in hBACs (Fig. 5A). Furthermore, prolonged induction increased the expression of PGC1α, PPARγ, CIDEA, FABP4, and UCP1 at both the mRNA (Fig. 5B) and protein levels (Fig. 5C). However, a decrease in the UCP1/FABP4 ratio indicated that the elevated expression of UCP1 was only partly due to the increased level of cell differentiation (Fig. 5B,C, right). The increased length of the induction period resulted in increased lipid droplet accumulation but reduced brown identity, suggesting that four days of induction was sufficient for BAC differentiation.

### Effects of adipogenic cocktail on lipid accumulation, functional properties and metabolic characteristics

To evaluate the efficacy of different primary BAC induction programs, we compared our induction program with other reported programs, as shown in Table 1. These induction schemes were labeled a, b, c, d, and e, sequentially. Groups a, b, and e increased the differentiation efficacy, as shown by ORO staining (Fig. 6A). Significantly higher gene expression levels of brown adipocyte markers were detected in cells in groups d and e (Fig. 6B). Remarkably, the UCP1 expression levels in these two groups were much higher relative to the levels of PPIA and FABP4. The changes in protein expression were consistent with those in mRNA expression (Fig. 6C).

To assess whether the upregulation of UCP1 gene and protein expression was functionally significant in adipocytes, we assessed mitochondrial function by measuring the OCR using an extracellular flux analyzer (Seahorse XF24 Extracellular Flux Analyzer) (Fig. 6D), with all data normalized to protein concentrations. The maximal respiration and UCP1-dependent respiration (Fig. 6E) were significantly (P < 0.001) highest in the BACs induced by protocol e within 2% BSA^27,43^, which sequestered the free fatty acids at three different time points. Taken together, these results confirmed that brown adipocytes induced by the program had significantly increased mitochondrial content and activity, important functional characteristics of BACs.

## Discussion

The thermogenic capacity of BAT makes it an attractive therapeutic target for weight loss and metabolic disorder improvement through energy expenditure. Indeed, human studies have shown that BAT activation is related to triglyceride clearance^44^ and glucose homeostasis^9^. Although these observations demonstrate the feasibility of BAT activation as an anti-obesity therapy, the underlying molecular mechanism and regulatory factors are not fully characterized. Much of our understanding of the characteristics, functions and molecular identity of BAT is still based on rodent models. Thus, there is an urgent need to develop a human classical brown adipocyte model and corresponding effective induction programs.

Primary hBAC culture is independent of exogenous immortalized gene^45^ or adipogenic gene transfection^46^, but limited by the difficulty (small numbers) of sample isolation from adult humans^15^. The primary hBACs from the interscapular region are considered the best *in vivo* BAT model. However, some deficiencies still need to be improved. First, we found that DMEM/F12 stimulated the adipogenic and thermogenic differentiation of BACs more effectively than DMEM (LG) and DMEM (HG). Although we found that FBS slightly promoted differentiation, it did not influence gene expression levels. Paradoxically, recent studies^22,47^ have shown that a high serum concentration is not required for the survival of human pre-adipocytes during adipogenesis and that exposure to serum actually inhibits differentiation. As previous studies have described^42,47,48^, FBS contains a complex array of protein components, such as growth factors, hormones, and amino acids. Moreover, the variations of FBS from different batches or corporations are not appropriate for drug research. Considering these issues, we recommend the use of the serum-free system for brown adipocyte induction.

INDO is commonly used for differentiating pre-adipocytes into brown/beige adipocytes in rodents^22^. In contrast, our results revealed that INDO could inhibit hBAC differentiation. Madsen L *et al*.^49^ also reported that INDO slightly attenuated UCP1 expression in the WT-1 cell model representing interscapular brown adipocytes. Accordingly, in some reports^16,32^, adding INDO into the induction cocktail of human pre-adipocytes did not improve the differentiation rate, as evaluated by ORO staining, even with a long induction period (7 days). The opposite effect of INDO on hBAC differentiation than mouse BAC differentiation may result from the species difference. Conversely, IBMX, ROG and DEX are essential components of the induction cocktail for promoting BAC differentiation. Based on current studies, we propose general concentrations of IBMX, DEX, and ROG for BAC induction, as shown in Table 1. IMBX (0, 0.25 mM, 0.5 mM) was demonstrated to promote hBAC differentiation and stimulate UCP1, PGC1α, CIDEA, PPARγ and FABP4 expression in a concentration-dependent manner. The promoted effect on the DEX-induced expression of BAT-related genes was not significantly different between 1 μM and 5 μM. The UCP1/FABP4 ratio was even lower at the higher concentration (5 μM) of DEX. The widely accepted role of glucocorticoids is to increase body mass and promote white pre-adipocyte maturation, resulting in obesity^50^. In contrast with white adipocytes, DEX treatment inhibited both mouse BAC differentiation and the energy expenditure of primary adipocytes^51^. Paradoxically, in our study, DEX was found to promote the differentiation and energy expenditure of hBACs, as measured by enhanced BAT-specific gene expression and basal metabolic activity^52^. This discrepancy may be due to the use of BAT from different species.

T3 is an essential factor for the induction and maintenance of brown fat cell characteristics^53^. The present study indicates that T3 treatment induced UCP-1 expression and mitochondrial biogenesis, resulting in the increased cellular OCR at a mimic plasma concentration (1 nM). Additionally, a protocol for introducing typical 3T3-L1 white adipocytes to differentiate into beige adipocytes used a high concentration (50–250 nM) of T3^54,55^. The T3 concentration in present study exerted similar effects in differentiating human multipotent adipose-derived stem cells^30^ into brown adipocytes.

Previous reports have demonstrated that concentrations of insulin lower than 430 nM might not be sufficient for BAT induction^38^. However, our findings revealed that a high concentration of insulin in the induction cocktail might be harmful for primary hBACs. We demonstrated that insulin concentrations in the range from 430 nM to 860 nM, rather than a certain concentration, were suitable for hBAC induction. A recent study also showed that brown adipocyte markers (UCP1, PGC1α), active mitochondrial numbers, oxygen consumption and respiratory capacity were decreased by chronic hyperinsulinemia^56^.

During BAC induction, the addition of d-biotin, apo-transferrin and pantothenate promoted BAC differentiation. This finding was consistent with similar results for the other kind of adipocyte^40^. Our observation demonstrates that four days of induction is sufficient for hBAC differentiation. However, prolonging the induction period could promote the expression of adipogenesis-related genes. Furthermore, metabolic characteristics were assessed to verify the results of gene levels. We found that brown adipocytes induced by program e exhibited increased UCP1 protein expression and UCP1-dependent OCRs.

In this article, we focused on optimizing the induction cocktail to improve hBAC differentiation levels. Additional reagents in the maintenance cocktail could further aggrandize thermogenic gene expression. We proposed an induction scheme adapted for hBACs using a basic induction cocktail consisting of critical factors: insulin, IBMX, DEX, ROG, and T3 in DMEM/F12. To improve hBAC differentiation, apo-transferrin, d-biotin and pantothenate acid are considered selective factors that could be added to the cocktail. Of course, it is possible that appropriately increasing the concentrations of basic induction factors or prolonging the induction time could partly improve the thermogenic capacity by promoting adipogenesis, especially after multiple passages. Our study provides an optimized induction method for primary BAC research on the activation of BAT to treat obesity and its metabolic consequences.
